# Fast generation of three-qubit Greenberger-Horne-Zeilinger state based on the Lewis-Riesenfeld invariants in coupled cavities

**DOI:** 10.1038/srep25707

**Published:** 2016-05-24

**Authors:** Xiao-Bin Huang, Ye-Hong Chen, Zhe Wang

**Affiliations:** 1 Department of Physics, Fuzhou University, Fuzhou 350002, China

## Abstract

In this paper, we propose an efficient scheme to fast generate three-qubit Greenberger-Horne-Zeilinger (GHZ) state by constructing shortcuts to adiabatic passage (STAP) based on the “Lewis-Riesenfeld (LR) invariants” in spatially separated cavities connected by optical fibers. Numerical simulations illustrate that the scheme is not only fast, but robust against the decoherence caused by atomic spontaneous emission, cavity losses and the fiber photon leakages. This might be useful to realize fast and noise-resistant quantum information processing for multi-qubit systems.

Entanglement is not only a key resource for quantum information processing (QIP)^1^,^2^, but also an essential ingredient for demonstrating quantum nonlocality^3^,^4^. Generally speaking, entanglement of multi-qubit is more useful for quantum applications and shows more clear nonclassical effects. For the case of three-qubit, there are two inequivalent classes of tripartite entanglement states, the Greenberger-Horne-Zeilinger (GHZ) state^4^ and the *W* state^5^. In contrast with the *W* state, the GHZ state provides a possibility to test quantum mechanics against local hidden theory without inequality^4^ and has practical applications in e.g., quantum secrete sharing^6^.

Thus, the manipulation of the GHZ state has attracted attention in recent years. A large number of theoretical and experimental proposals have been proposed for producing this entangled state^7^,^8^,^9^,^10^,^11^,^12^,^13^,^14^,^15^. However, most of the previous schemes either require a relatively long operation time (adiabatic passage) or need to control the interaction time accurately (quantum Zeno dynamics). These make the schemes are difficult to implement in experiments. Therefore, in recent year, a main goal in QIP is to overcome the drawbacks and combine advantages of adiabatic passage and quantum Zeno dynamics (QZD)^16^. Fortunately, a famous technique named “shortcuts to adiabatic passage” (STAP)^17^,^18^,^19^,^20^,^21^,^22^,^23^,^24^,^25^,^26^,^27^, which can fast and robustly generate entangled states, makes the above goal become true. This technique is related on adiabatic passage but successfully breaks the time limit in an adiabatic process. It can obtain the same final populations with adiabatic but is just a fast adiabatic-like process which is not really adiabatic. The shortcut techniques mainly include counter-diabatic driving (CD)^17^,^18^,^19^ or, equivalently, transitionless quantum driving^20^,^21^ and inverse engineering based on Lewis-Riesenfeld invariants^25^,^26^,^27^. Among these shortcut techniques, the invariant-based method has been applied to accelerate the adiabatic processes for trap expansion or compressions^26^,^27^ and atomic transport^28^,^29^,^30^. In fact, CD and invariant-based engineering can be shown to be potentially equivalent methods by properly adjusting the reference Hamiltonian^31^.

In the last several years, many schemes have been proposed in theoretically and experiments based on STAP^26^,^27^,^28^,^29^,^30^,^31^,^32^,^33^,^34^,^35^,^36^,^37^,^38^,^39^,^40^,^41^,^42^,^43^,^44^,^45^,^46^,^47^,^48^. Among these schemes, del Campo *et al*. first presented the multi-qubit shortcuts scheme in ref. 32. After that, many innovative schemes have been presented, i.e., Chen *et al*. constructed shortcuts to perform fast and noise-resistant populations transfer in multi-particle systems by combining “LR invariants” with “QZD”^33^. However, most of the above STAP schemes only focus on the single cavity situation, which is still a challenge to manipulate a large number of qubits. The coupled-cavity systems^49^,^50^,^51^,^52^,^53^,^54^,^55^ are considered as a suitable candidate for the solution of the above deficiency and for the construction of a practical quantum network. In view of that, we wonder if it is possible to construct STAP for the generation of multi-qubit entanglement in coupled cavity systems. And this would be an interesting direction in quantum state engineering.

In this paper, we construct STAP to fast generate GHZ state in spatially separated cavities by combining “LR invariants” with “QZD”. Our scheme has the following advantages: (1) the operation time required for the creation of the GHZ state is relatively short. (2) This scheme is not only robust against parameters fluctuation in the experimental, but need’t accurately control the operation time. (3) Numerical simulations show that the decoherences such as atomic spontaneous emission, cavity losses and the fiber photon leakages have little influence on this scheme. (4) Individual addressing becomes relatively easy in coupled cavity systems.

This paper is structured as follows. In Sec. II, we give a brief description of the preliminary theory about LR invariants and QZD. In Sec. III, we construct STAP based on the invariant-based inverse engineering and show how to use STAP to fast generate GHZ state. In Sec. IV, we give the numerical simulations and discussions for our schemes. A discussion on experimental feasibility and a summary appear in Sec. V.

## Preliminary Theory

### Lewis-Riesenfeld invariants

Firstly, we briefly describe LR invariants theory^25^. We consider a time-dependent quantum system whose Hamiltonian is *H*(*t*). Associated with the Hamiltonian there are time-dependent Hermitian invariants of motion *I*(*t*) that satisfies


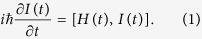


The solution of the time-dependent Schrödinger equation 
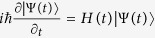
 can be expressed by a superposition of invariant *I*(*t*) dynamical modes |Φ_*n*_(*t*)〉


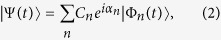


where *C*_*n*_ is the *n*th constant, |Φ_*n*_(*t*)〉 is the *n*th eigenvector of *I*(*t*) and the corresponding real eigenvalue is *ς*_*n*_. The Lewis-Riesenfeld phases *α*_*n*_ fulfill


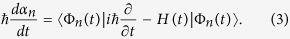


### Quantum Zeno dynamics

Next, we give a brief review of the quantum Zeno dynamics. According to ref. 16, we know the main features of the QZD can be obtained by making use of a continuous coupling. We consider a system which is governed by the Hamiltonian


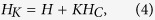


where *H* is the Hamiltonian of the quantum system to be studied; *H*_*C*_ can be viewed as an additional interaction Hamiltonian which plays the role of measurement; *K* is the coupling constant. When *K* → ∞, the subsystem of interest is dominated by the evolution operator


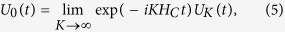


which can be shown to have the form


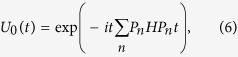


where *P*_*n*_ is one of the eigenprojections of *H*_*C*_ with eigenvalues *η*_*n*_(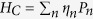). So the whole system is governed by the limiting evolution operator


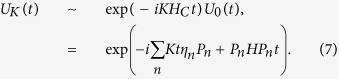


## Fast Preparation of GHZ State by Combining “Lewis-Riesenfeld Invariants” with “Quantum Zeno Dynamics”

**Figure 1:**
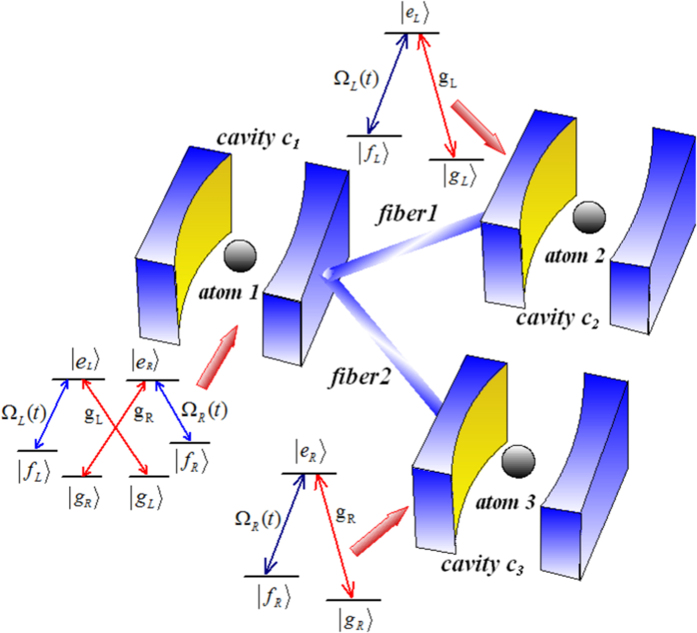
Experimental setup and level configuration for each atom.

As shown in Fig. 1, a six-level atom and two Λ-type atoms are trapped in three distant optical cavities coupled by two short optical fibers. The cavity *C*_1_ is bimodal-mode, the cavities *C*_2_ and *C*_3_ are single-mode. The first atom has two degenerate excited states |*e*_*L*_〉 and |*e*_*R*_〉, two degenerate ground states |*g*_*L*_〉 and |*g*_*R*_〉, and two intermediate states | *f*_*L*_〉 and | *f*_*R*_〉. The others atoms have a degenerate excited state |*e*_*L*_〉_2_ (|*e*_*R*_〉_3_), a degenerate ground state |*g*_*L*_〉_2_ (|*g*_*R*_〉_3_) and a intermediate state | *f*_*L*_〉_2_ (|* f*_*R*_〉_3_). The transitions |* f*_*L*_〉_1(2)_ ↔ |*e*_*L*_〉_1(2)_ and | *f*_*R*_〉_1(3)_ ↔ |*e*_*R*_〉_1(3)_ are resonantly driven through classical laser fields with time-dependent Rabi frequency Ω_*L*_(*t*) and Ω_*R*_(*t*), respectively. The atomic transitions |*g*_*L*_〉_1(2)_ ↔ |*e*_*L*_〉_1(2)_ and |*g*_*R*_〉_1(3)_ ↔ |*e*_*R*_〉_1(3)_ resonantly couple to the left-circularly and right-circularly polarized mode of cavities with coupling constants *g*_*L*_ and *g*_*R*_, respectively.

In the short-fiber limit, i.e., 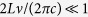 (where *L* denotes the fiber length, *c* denotes the speed of light, and *ν* denotes the decay of the cavity field into a continuum of fiber mode), only one resonant fiber mode interacts with the cavity mode^56^. In the interaction picture, the total Hamiltonian is


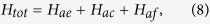


where


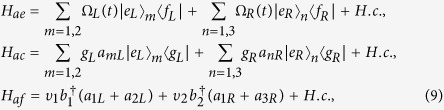


here *a*_*mL*_ (*a*_*nR*_) denotes annihilation operator for the left-(right-) circularly polarized mode of the *m* (*n*) cavity, 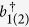 denotes the creation operator for the *f*_1,(2)_ fiber. The states of the three qubits are represented by {|*g*_*L*_〉, |*g*_*R*_〉}, {|*f*_*L*_〉, |*g*_*L*_〉}, and {| *f*_*R*_〉, |*g*_*R*_〉}. For the sake of simplicity, we assume *g*_*L*_ = *g*_*R*_ = *g*, and *υ*_1_ = *υ*_2_ = *υ*. If we assume the initial state of the system is | *f*_*L*_〉|*g*_*L*_〉|*g*_*R*_〉|00〉_*c*1_|0, 0〉_*c*2,*c*3_|0, 0〉_*f*1,*f*2_, the whole system evolves in the subspace 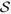 spanned by:


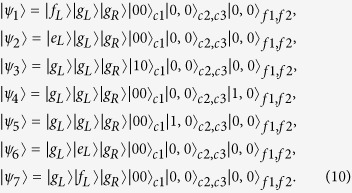


Under the Zeno condition Ω_*L*_, Ω_*R*_ ≪ *g*, *υ*, the Hilbert subspace 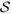 is split into five Zeno subspaces according to the degeneracy of eigenvalues of the Hamiltonian *H*_*im*_ = *H*_*ac*_ + *H*_*af*_


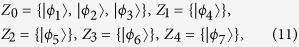


where the eigenstates of *H*_*im*_ are


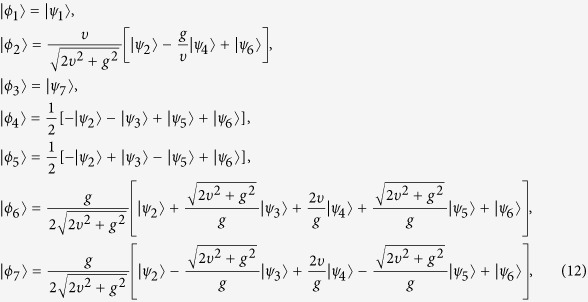


the corresponding eigenvalues are *ς*_0_ = 0, *ς*_1_ = *g*, *ς*_2_ = −*g*, 
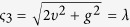
, 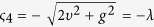 with the projections (*k* = 0, 1, 2, 3, 4)


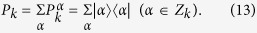


Under the Zeno condition, according to the ref. 16, the Hamiltonian of the current system is approximately dominated by


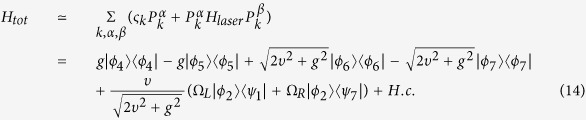


As the initial state is |*ψ*_0_〉 = | *f*_*L*_〉|*g*_*L*_〉|*g*_*R*_〉|00〉_*c*1_|0, 0〉_*c*2,*c*3_|0, 0〉_*f*1,*f*2_, the system will always evolve in the Zeno subspace *Z*_0_, and the effective Hamiltonian of the current system reduces to


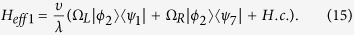


On the other hand, if the initial state is 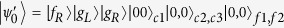, the whole system evolves in the subspace 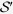 spanned by:


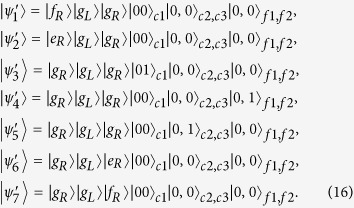


Then, performing similar processes from Eq. (11) to Eq. (14), we obtain the effective Hamiltonian of the current system


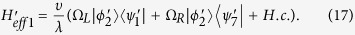


According to the above analysis, we can draw a conclusion that the states | *f*_*L*_〉|*g*_*L*_〉|*g*_*R*_〉|00〉_*c*1_|0, 0〉_*c*2,*c*3_|0, 0〉_*f*1,*f*2_ and | *f*_*R*_〉|*g*_*L*_〉|*g*_*R*_〉|00〉_*c*1_|0, 0〉_*c*2,*c*3_|0, 0〉_*f*1,*f*2_ do not interact with each other during the evolution because they evolve in different Zeno invariant subspaces, respectively. The global phase of the quantum Zeno dynamics does not play any role in the evolution because of the resonant interaction and the symmetry structure. If the initial state is prepared in the state 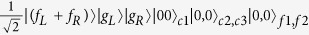, the system evolves in Zeno subspace spanned by 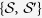, and the effective Hamiltonian of the current system becomes


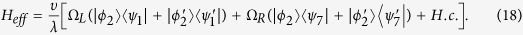


Then we use six orthogonal vectors


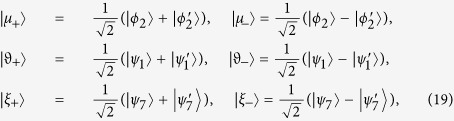


to rewrite the Hamiltonian in Eq. (18) as


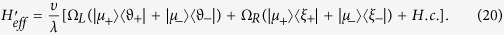


It is obvious that when the initial state is |*ϑ*_+_〉, the terms containing |*μ*_−_〉, |*ϑ*_−_〉 and |*ξ*_−_〉 are negligible because they are decoupled to the time evolution of initial state. Then we can obtain the final effective Hamiltonian


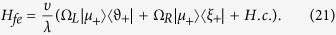


In order to speed up preparation of target state by using the dynamics of invariant-based inverse engineering, we need to find out the Hermitian invariant operator *I*(*t*), which satisfies 
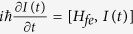
. As the Hamiltonian *H*_*fe*_ possesses SU(2) dynamical symmetry, so *I*(*t*) can be easily given by^33^,^34^


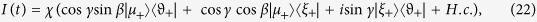


where *χ* is an arbitrary constant with units of frequency to keep *I*(*t*) with dimensions of energy, *γ* and *β* are both time-dependent auxiliary parameters. Through solving the relation 
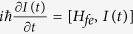
, Ω_*L*_ and Ω_*R*_ are obtained


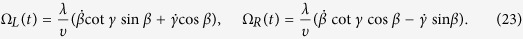


The eigenstates of the invariant *I*(*t*) are


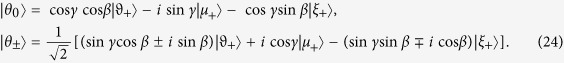


The solution of Schrödinger equation 
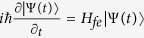
 can be written with the eigenstates of *I*(*t*) as


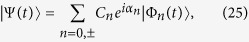


where as mention in Eq. (3), here should be: *α*_*n*_(*t*) are the LR phases mentioned in section II, and the form of *α*_*n*_ is


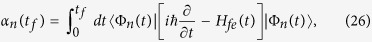


where *t*_*f*_ is the total interaction time. Similarly, in our case *α*_0_ = 0, and


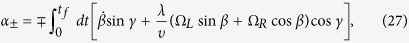


In order to generate GHZ state, we choose the parameters as


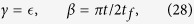


where 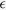 is a time-independent small value and *t*_*f*_ is the interaction time. Then, we obtain


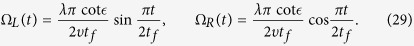


In the present case, when *t* = *t*_*f*_,


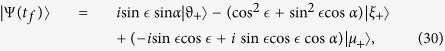


where 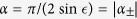. When we choose *α* = 2*Nπ*(*N* = 1, 2, 3 ···), 
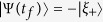
. Therefore, the whole system quickly evolves from the initial state |*ϑ*_+_〉 to the final state −|*ξ*_+_〉. That is to say, the three-qubit GHZ state can be obtained


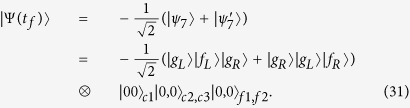


## Numerical Simulations and Discussions

In this section, we make the numerical simulations for the GHZ state by numerically solving the Schrödinger equations. We also discuss the influence of the decoherence caused by atomic spontaneous emission, cavity losses and the fiber photon leakages.

**Figure 2:**
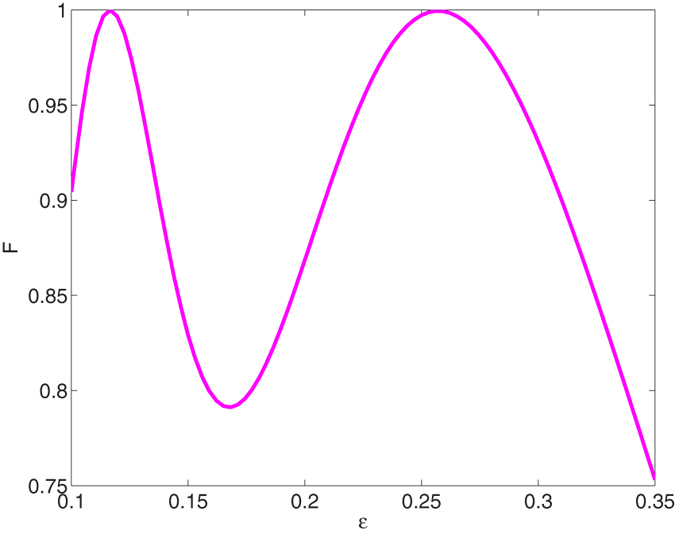
The fidelity of the GHZ state versus parameter 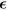 when *gt*_*f*_ = 30 and *υ* = *g*.

**Figure 3:**
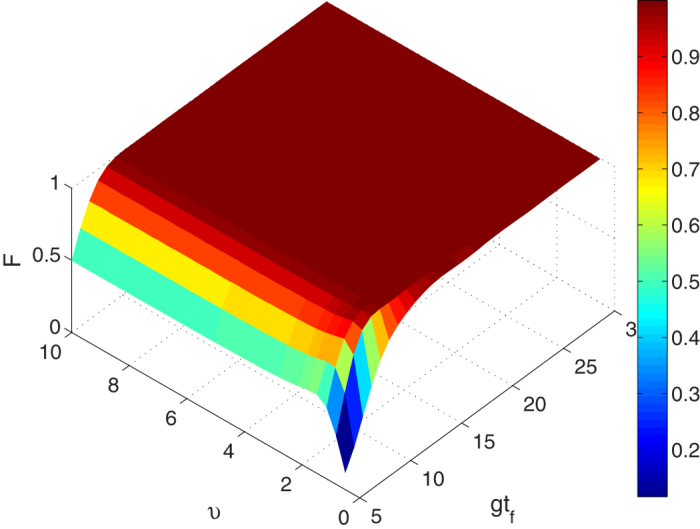
The fidelity of the GHZ state versus parameters *υ* and *gt*_*f*_.

**Figure 4:**
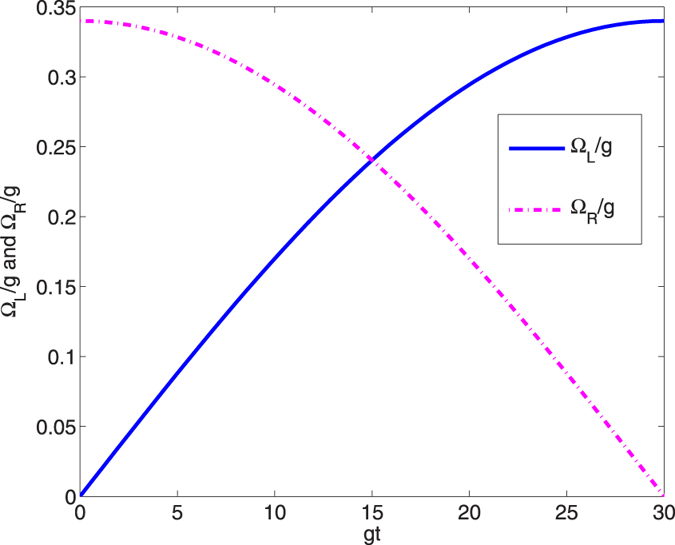
Dependence on *gt* of Ω_*L*_/*g* and Ω_*R*_/*g*.

**Figure 5:**
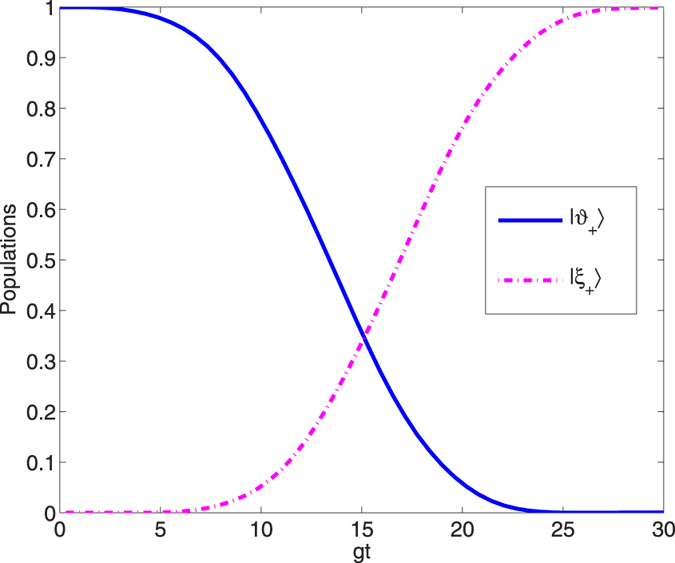
Dependence on *gt* of the populations for the initial state |*ϑ*_+_〉 and the target state |*ξ*_+_〉.

**Figure 6:**
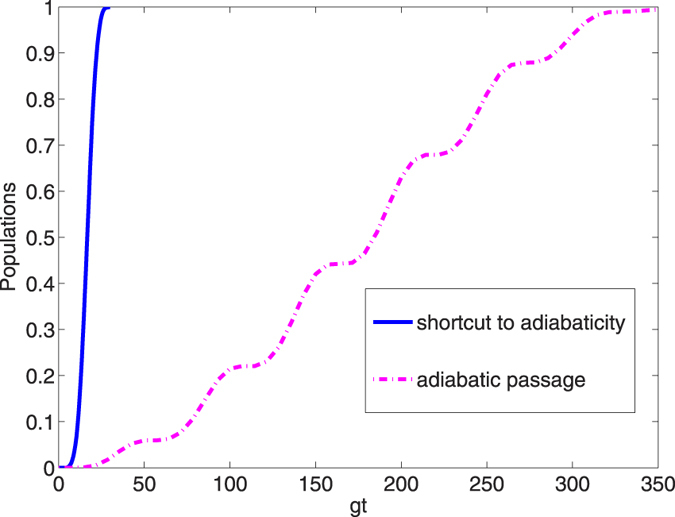
The comparison of the operation times required for achieving the target state via the adiabatic method with those via the present STAP method.

Firstly, we plot the fidelity *F* of the target state |Ψ(*t*_*f*_)〉 versus the value of 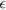 and *gt*_*f*_ in Fig. 2, where the fidelity of the state is defined as *F* = |〈*ψ*|*ρ*(*t*_*f*_)|*ψ*〉|. Figure 2 shows that the ideal value of 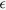 for the highest fidelity is slightly different from the condition 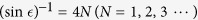 in ref. 34. The reason for this difference has been discussed in ref. 33 in detail: in the present case, the Zeno condition is satisfied but not very ideally because speeding up the system requires relatively large laser intensity. Therefore, under the premise that the interaction time for the entangled operation is short. In order to satisfy the Zeno condition as well as possible, the parameters should be chosen as 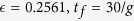. Meanwhile, we analyse the relation between the cavity-fibre coupling *υ* and the interaction time *t*_*f*_ since *υ* plays a very important role in the evolution. The fidelity *F* versus *υ* and *gt*_*f*_ is shown in Fig. 3 with 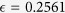. Figure 3 shows that the increasing value of *υ* does not help to shorten the interaction time. The reason is that the relation between the coupling *υ* and the amplitude of the laser pulses Ω_0_ at that time was not taken into consideration. In fact, shortening the time requires increasing the amplitude of the laser pulses. The amplitude of the laser pulses in Eq. (29) inverses the proportion to the coupling *υ*, i.e., the amplitude is smaller, the interaction time is longer. Consequently, it is wisely to choose *υ* = *g* in our method. Moreover, Fig. 3 shows that in the present case, the shortest interaction time required for an ideal population transfer from the initial state |*ϑ*_+_〉 to target state |Ψ(*t*_*f*_)〉 is only about 9/*gs*. It means that the entangled state can be fast generated. In Fig. 4, we plot the scaled Rabi frequencies Ω_*L*_(*t*)/*g* and Ω_*R*_(*t*)/*g* versus *gt* when 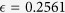, *gt*_*f*_ = 30 and *υ* = *g*. The amplitude of the laser pulse Ω_0_ is 1.05 *g* which meets the conditions mentioned above, and such an intensity is safe to assume linear optic models. The population curves of state |*ϑ*_+_〉 and |*ξ*_+_〉 versus *gt* are depicted Fig. 5. From Fig. 5, we can see a perfect population transfer from the initial state |*ϑ*_+_〉 to the target state |*ξ*_+_〉 after the whole evolution, and a GHZ state can be generated according to Eq. (30). We contrast the interaction time required for achieving the target state via an adiabatic process with the present STAP method in Fig. 6. We can see from Fig. 6, the present STAP method effectively shortens the interaction time of the adiabatic method.

**Figure 7:**
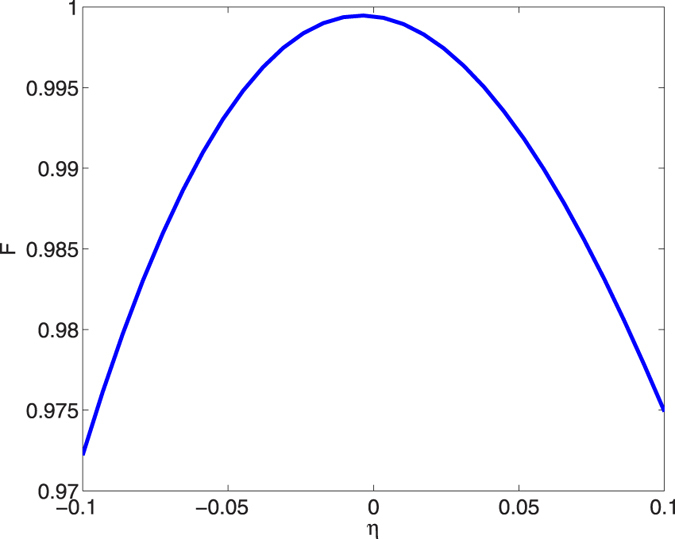
The fidelity *F* versus the deviation of the classical Rabi frequencies with the deviation parameter *η* = *δ*Ω_*L*_/Ω_*L*_ = *δ*Ω_*R*_/Ω_*R*_, and other parameters 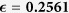, *t*_*f*_ = 30/*g* and *υ* = *g*.

**Figure 8:**
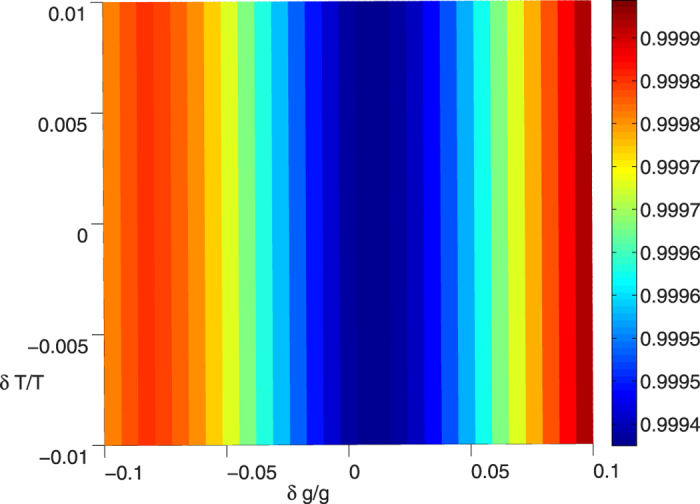
The fidelity *F* versus the deviation of *δg*/*g* and *δT*/*T* when *T* = *t*_*f*_ = 30/*g*, and other parameters 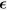 = 0.2561 and *υ* = *g*.

To check the robustness against to the variation of different parameters, in Fig. 7, we calculate the fidelity versus the deviation of the classical Rabi frequencies with the deviation parameter *η* = *δ*Ω_*L*_/Ω_*L*_ = *δ*Ω_*R*_/Ω_*R*_. It is apparent that the fidelity of prepared state |Ψ(*t*_*f*_)〉 is always higher than 97.2%, when *η* = ±0.1 with 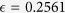 and *t*_*f*_ = 30/*g*. Furthermore, The fidelity versus the deviation *δT*/*T* and *δg*/*g* is shown in Fig. 8. As one can see from Fig. 8 that the the fidelity is very insensitive to the variation of *g* and *t*_*f*_ since it always higher than 99.4% under condition *T* = *t*_*f*_ = 30/*g*. Thus, we can draw a conclusion that our scheme is robust against variation of the parameters, such as Ω_*L*_, Ω_*R*_, *g*, *t*_*f*_ and so on.

**Figure 9:**
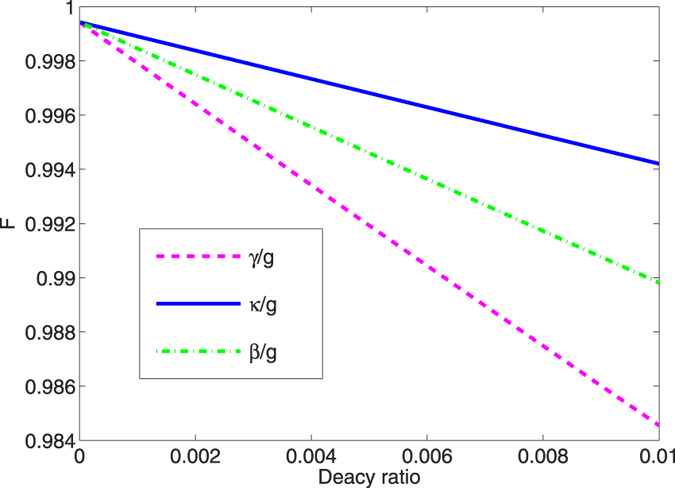
The fidelity *F* versus the decoherence parameters *γ*/*g*, *κ*/*g* and *β*/*g* with the parameters 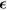 = 0.2561, *t*_*f*_ = 30/*g* and *υ* = *g*.

**Figure 10:**
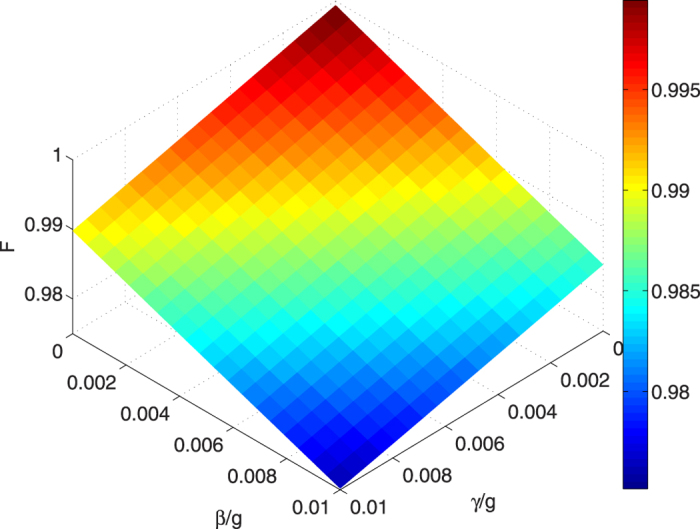
Dependences on the decoherence parameters *γ*/*g* and *β*/*g* of the fidelity of the GHZ state via STAP with the parameters 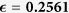, *t*_*f*_ = 30/*g* and *υ* = *g*.

Next, we will investigate the influence of various decoherence caused by the atomic spontaneous emission, cavity losses and the fiber photon leakages. The master equation of the whole system reads


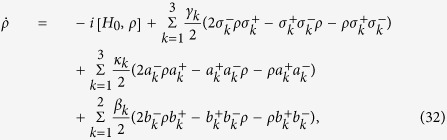


where *γ*_*k*_ denotes the atomic spontaneous decay rate of the *k*th atom; *κ*_*k*_ and *β*_*k*_ denote the decay rates of the *k*th cavity and fiber, respectively. For simplicity, we set *γ*_1_ = *γ*_2_ = *γ*_3_ = *γ*, *κ*_1_ = *κ*_2_ = *κ*_3_ = *κ* and *β*_1_ = *β*_2_ = *β*. The master equation can be numerically solved in the subspace 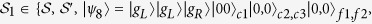 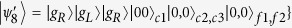. We plot the fidelity versus the dimensionless parameters *γ*/*g*, *κ*/*g* and *β*/*g* in Fig. 9. We can see from Fig. 9 that the fidelity of GHZ state is almost unaffected by the cavity decay, since the probability of the system evolving beyond the Zeno subspace is very small. Thus, The atomic spontaneous emission and the fiber loss become the main sources of decoherence. As shown in Fig. 10, the fiber loss and the atomic spontaneous emission has a little influence on the fidelity, the GHZ state has a high fidelity 97.52% when we set *γ* = *β* = 0.01 *g*. This means that the proposed scheme is robust against the decoherence since the fidelity nearly never varied while the parameters are diversifications.

## Experimental Feasibility and Conclusions

Finally, let us consider the experimental feasibility of the proposed scheme. The bimodal cavity can sustain two degenerate modes with the same coupling strengths in the experiment reported by ref. 57 and the required atomic level configuration can be implemented with ^40^Ca^+^^57^ and ^198^Hg^+^^58^. The Zeeman substates |−1/2, *P*_1/2_〉 and |1/2, *P*_1/2_〉 act as the states |*e*_*L*_〉 and |*e*_*R*_〉, respectively. The substates |1/2, *S*_1/2_〉 and |−1/2, *S*_1/2_〉 act as the states |*g*_*L*_〉 and |*g*_*R*_〉, respectively. The substates |−3/2, *D*_3/2_〉 and |1/2, *D*_3/2_〉 equally couple to |−1/2, *P*_1/2_〉 by the classical fields, while |−1/2, *D*_3/2_〉 and |3/2, *D*_3/2_〉 equally couple to |1/2, *P*_1/2_〉. If the atom is initially in the symmetric superposition state 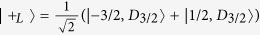 or 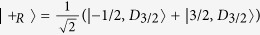, the classical fields induce the effective transition |+_*L*_〉 ↔ |*e*_*L*_〉 or |+_*R*_〉 ↔ |*e*_*R*_〉. Therefore, the superposition states |+_*L*_〉 and |+_*R*_〉 can act as the states | *f*_*L*_〉 and | *f*_*R*_〉, respectively. Furthermore, a set of cavity quantum electrodynamics (QED) parameters (*λ*, *γ*, *κ*)/2*π* = (750, 2.62, 3.5) MHz is predicted to be available^59^, with the cavity wavelength is about 850 nm. The fiber loss at the 852 nm wavelength is 2.2 dB/km^60^, corresponding to a fiber decay rate *β* = 0.152 MHz. In this condition, the fidelity of the GHZ state is 99.81% in our scheme.

In summary, we have proposed an efficient theoretical scheme to fast generate a GHZ state for three atoms trapped in coupled cavities linked by optical fibers based on STAP by combining “LR invariants “ with “QZD”. The influences of the decoherence such as atomic spontaneous emission, cavity losses and the fiber photon leakages are numerically studied. Numerical simulations demonstrate that our scheme is not only fast, but also robust against the decoherence. Additionally, numerical simulations also demonstrate our scheme is robust against variation of the parameters, such as Ω_*L*_, Ω_*R*_, *g*, *υ*, *t*_*f*_ and so on. We believe the shortcut method is useful to realize fast and noise-resistant quantum information processing for multi-qubit systems.

## Additional Information

**How to cite this article**: Huang, X.-B. *et al*. Fast generation of three-qubit Greenberger-Horne-Zeilinger state based on the Lewis-Riesenfeld invariants in coupled cavities. *Sci. Rep*. **6**, 25707; doi: 10.1038/srep25707 (2016).
