# Corrigendum: Fast generation of three-qubit Greenberger-Horne-Zeilinger state based on the Lewis-Riesenfeld invariants in coupled cavities

**DOI:** 10.1038/srep29149

**Published:** 2016-07-20

**Authors:** Xiao-Bin Huang, Ye-Hong Chen, Zhe Wang

Scientific Reports 6: Article number: 2570710.1038/srep25707; published online: 05242016; updated: 07202016

The Acknowledgements section in this Article is incomplete.

“This work was supported by the National Natural Science Foundation of China under Grants No. 11404061, and the Natural Science Foundation of Fujian Province under Grant No. 2016J01018”.

should read:

“This work was supported by the National Natural Science Foundation of China under Grants No. 11404061, and the Natural Science Foundation of Fujian Province under Grant No. 2016J01018, and the Major State Basic Research Development Program of China under Grant No. 2012CB921601”.

